# Characteristics of 1946 Cases of POEMS Syndrome in Chinese Subjects: A Literature-Based Study

**DOI:** 10.3389/fimmu.2019.01428

**Published:** 2019-06-21

**Authors:** Yong Wang, Li-Bo Huang, Yi-Hua Shi, Huan Fu, Zhen Xu, Guo-Qing Zheng, Yan Wang

**Affiliations:** Department of Internal Medicine, the Second Affiliated Hospital and Yuying Children's Hospital of Wenzhou Medical University, Wenzhou, China

**Keywords:** POEMS syndrome, Chinese population, clinical manifestations, peripheral neuropathy, plasma cell dyscrasia

## Abstract

POEMS syndrome is a rare paraneoplastic disorder characterized secondary to a rare plasma cell dyscrasia. Here, we aimed to analyze the clinical characteristics of large sample cases of POEMS in Chinese subjects through making a review of the Chinese literature. Four databases were electronically searched from inception until October 2016. Case reports and case series were identified. Six hundred studies with 1946 participants were identified. The first case was reported in 1986, and the number of reported cases peaked in 2009 and 2010. The top seven provinces on the number of reported cases were in the south-east area of China. The top three departments on the number of published papers and reported cases were ordinally department of Neurology, Hematology, and Endocrinology. The ratio of male to female was about 2.23. The range of age onset was from 10 to 81 years with the mean age of 46.39 (SD, 12.10 years). The initial symptoms of POEMS with peripheral neuropathy, edema and effusions, endocrinopathy, skin changes, and organomegaly accounted for 60.44, 15.72, 9.87, 8.05, and 2.13%, respectively, and subsequently acquired above symptoms as the prevalence was 99.49, 81.91, 75.56, 77.08, and 83.09%, respectively. The present study would help to understand the clinical presentations of POEMS syndrome in the Chinese population.

## Introduction

POEMS syndrome, also less frequently called Crow-Fukase syndrome (1) or Takatsuki syndrome (2), is a rare paraneoplastic disorder secondary to an underlying plasma cell neoplasm (3). The memorable acronym was coined by Bardwick et al. (4), which refers to several main features of the syndrome: polyneuropathy, organomegaly, endocrinopathy, monoclonal plasma cell disorder, and skin changes. Additional important features include papilledema, extravascular volume overload, sclerotic bone lesions, thrombocytosis/erythrocytosis, elevated VEGF levels, a predisposition toward thrombosis, and abnormal pulmonary function tests (5). The first report of what is now called POEMS syndrome was Scheinker's autopsy case in 1938 (6). A syndrome distinct from multiple myeloma-associated neuropathy began to be recognized since Crow's description of two patients with osteosclerotic plasmacytomas in 1956 (7). Since then, a number of cases have been reported. The initial case series reports came from Japan, where 109 and 102 cases of POEMS syndrome were reported in 1983 (2) and in 1984 (1), respectively. A national survey conducted in Japan in 2012 indicated a prevalence of approximately 0.3 per 100,000 (8). Over the years, some case series also were reported in France (9), the United States (10), India (11), and China (12, 13).

The complete understanding of the pathogenesis of POEMS has not yet been achieved, but significant advances have been made. The interplay of several cytokines involved in angiogenesis and microvascular permeability might be significant in the pathogenesis (15, 16). To date, VEGF is the cytokine of what correlates best with this disease activity (14, 17–21). However, the mixed results of anti-VEGF therapy suggested that it may not be the driving force of the disease (22–24). Indeed, now it is widely accepted that VEGF is probably a downstream mediator of a paraneoplastic syndrome rather the pathogenic initiating factor (25). The role of B-cell dyscrasia was also highlighted in the pathogenesis of this disease (26). POEMS syndrome could be considered as a monoclonal gammopathy of clinical significance whereby all the damages are linked to a small toxic clone, which produces a monoclonal immunoglobulin (27, 28). This toxic protein seems to be a monoclonal lambda light chain encoded by only two lambda light chain variable genes (IGVL1), which is able to drive the synthesis of huge amounts of VEGF by an unknown mechanism (29–32).

In China, according to a case series reported by Li et al. (12) the percentage of misdiagnosis was 85%, and the median survival time was only 5–7 years if without successful treatment. The rarity and the complicated clinical manifestations of POEMS syndrome make accurate diagnosis difficult because seemingly disparate signs and symptoms must be linked to make the diagnosis (10). Although many cases have been reported in China, there were no large studies reviewing the characteristics of Chinese patients with POEMS syndrome. In addition, previous studies (12, 13, 33–35) were lack of information about initially and subsequently acquired clinical features and epidemiological characteristics of POEMS. Thus, in the present review of the Chinese literature, we identified a total of 1946 case of POEMS syndrome patients in China from 1986 to 2016 and systematically analyzed their epidemiological and clinical features.

## Methods

### Database and Search Strategies

We searched Chinese National Knowledge Infrastructure (CNKI), VIP Journals Database, Wanfang Database and Chinese Biomedical Databse (CBM) for POEMS syndrome of Chinese subjects from inception to October 31th 2016. Conference proceedings, dissertations, and references of previously published reviews were also searched manually for additional relevant studies. The following search strategy was used for CNKI and was modified to suit other databases.

1. POEMS
2. Crow-Fukase
3. Takatsuki
4. OR/1–3
5. Syndrome
6. 4 AND 5

### Inclusion and Exclusion Criteria

The inclusion criteria are prespecified as follows: (1) the case reports or series reports of POEMS syndrome were included; (2) all patients with POEMS syndrome met the diagnostic criteria proposed by Nakanishi et al. (1) or Dispenzieri et al. (10); (3) Chinese patients were the subjects of included studies (4) there are extractable report data in each study. The exclusion criteria met one of the following items: (1) duplicated publication; (2) review or abstract; (3) without extractable data.

### Data Extraction and Classification

The following information were extracted: title, author, publisher, year and department of publication; type of research design; the number of POEMS patients; gender and sex ratio; age of onset; clinical manifestations including polyneuropathy, organomegaly, endocrinopathy, skin changes, edema or effusion, and other manifestations; auxiliary examinations: laboratory examination, imaging examination, lumbar puncture, electromyography, and bone marrow aspiration; treatment methods and prognosis and follow-up.

### Data Management

All data were extracted using ACCESS 2016. SPSS 16.0 software was used for statistical analysis.

## Results

### Study Selection

**Figure 1:**
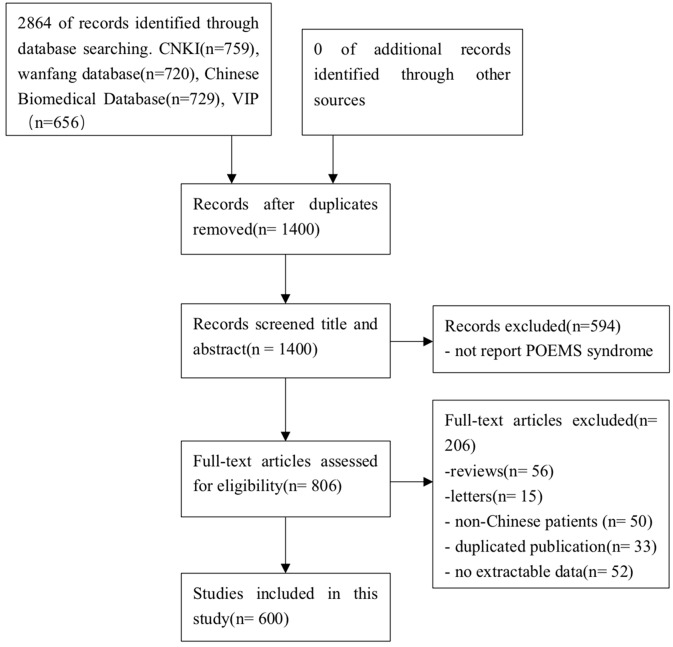
PRISMA 2009 flow diagram. A total of 2,864 potentially relevant hints were retrieved. After removal of duplicates, 1,400 records remained. After initial screening based on titles and abstracts, 594 articles were excluded for they did not report POEMS syndrome. Of the remaining 806 articles subjected to full-text reading, 206 articles were excluded in following reasons: 56 articles were reviews; 15 articles were letters; 50 articles reported non-Chinese patients; 52 articles did not provide extractable data; 33 articles were duplicated publication. Ultimately, 600 articles were included in present study.

A total of 2,864 potentially relevant hint were retrieved. After removal of duplicates, 1,400 records remained. After initial screening based on titles and abstracts, 594 articles were excluded for they did not report POEMS syndrome. Of the remaining 806 articles subjected to full-text reading, 206 articles were excluded in following reasons: 56 articles were reviews; 15 articles were letters; 50 articles reported non-Chinese patients; 52 articles did not provide extractable data; 33 articles were duplicated publication. Ultimately, 600 articles were included in present study. The screening process was summarized in a flow diagram (Figure 1).

### Characteristics of Included Studies

Six hundred studies with 1,946 individuals were identified. Of which, 401 studies were single case report and 199 studies were case series report. Five hundred and ninety-one studies were published in Chinese, and nine studies were published in English.

#### Year Distribution of Published Papers and Reported Cases

**Figure 2:**
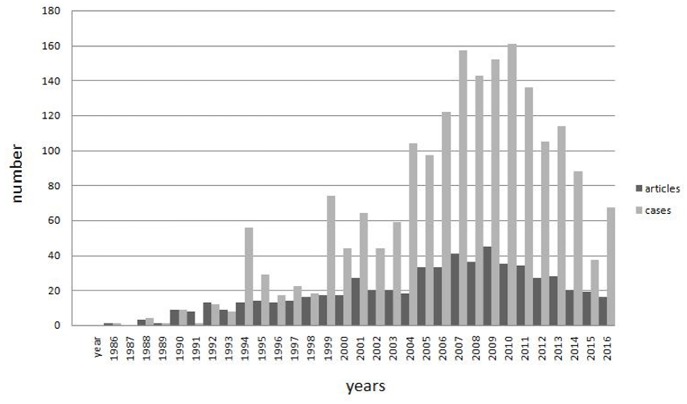
Year distribution of published papers and reported cases. In china, the first patient with clinical features similar to POEMS syndrome was reported in 1986. No new case was reported until 1988. From 1988 to 2010, the number of published papers and reported cases increased in fluctuation and peaked in 2009 and 2010, respectively. The number of patients was reported over 50 in 1994 and over 100 in 2004, reaching 161 in 2010 and declining gradually.

In China, the first patient with clinical features similar to POEMS syndrome was reported in 1986 (36). No new case was reported until 1988. From 1988 to 2010, the number of published papers and reported cases increased in fluctuation and peaked in 2009 and 2010, respectively. The number of patients was reported over 50 in 1994 and over 100 in 2004, reaching 161 in 2010 and declining gradually (Figure 2).

#### Regional Distribution of Published Papers and Reported Cases

**Figure 3:**
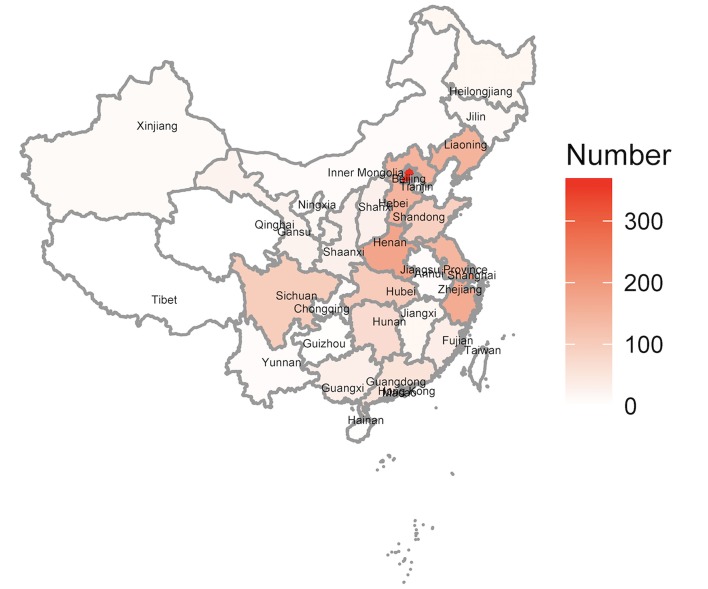
Regional distribution of reported cases. The top seven provinces on the number of reported cases were ordinally Beijing, Shanghai, Henan, Zhejiang, Liaoning, Hebei, and Jiangsu. There was no paper or case reported in Tibet, Taiwan, Hong Kong, and Macao.

The top seven provinces on the number of published papers were ordinally Beijing, Shanghai, Jiangsu, Zhejiang, Hebei, Shandong, and Guangdong. They published 56.18% of all papers. The top seven provinces on the number of reported cases were ordinally Beijing, Shanghai, Henan, Zhejiang, Liaoning, Hebei, and Jiangsu. These provinces reported about 66.32% of all cases, and each of them reported more than 100 cases. There was no paper or case reported in Tibet, Taiwan, Hong Kong, and Macao (Figure 3).

#### Department Distribution of Published Papers and Reported Cases

**Figure 4:**
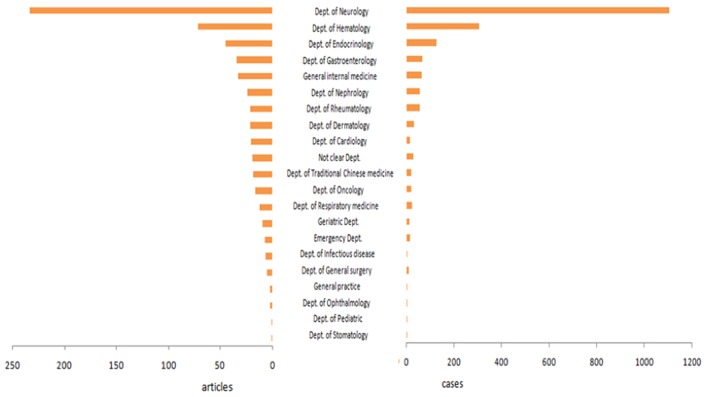
Department distribution of published papers and reported cases. The top three departments on the number of published papers and reported cases were ordinally department of Neurology, department of Hematology, and department of Endocrinology.

The top three departments on the number of published papers and reported cases were ordinally department of neurology, department of hematology, and department of endocrinology. In the present study, 58.16% of papers and 78.72% of cases were reported by above three departments. The patients reported by Neurology accounted for 53.73%. The rest of the papers and cases were reported by other areas, which account for 41.84 and 21.28%, respectively (Figure 4).

### General Information of Reported Cases

#### Gender Distribution

**Figure 5:**
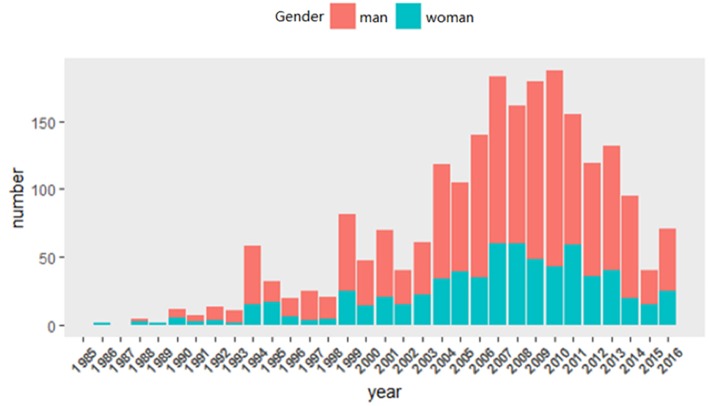
Gender distribution. A total of 1,946 cases were included in this study. There were 1,344 (69.06%) men and 602 (30.94%) women, and the ratio of male to female was about 2.23. From 1900 to 1993, the ratio of male to female was approximately 1:1, but there were few reported cases. Over 50 cases were reported in 1994, and males outnumbered females in this year. From then on, the ratio of male to female was over 1.

A total of 1,946 cases were included in this study. There were 1,344 (69.06%) men and 602 (30.94%) women, and the ratio of male to female was about 2.23. From 1900 to 1993, the ratio of male to female was approximately 1:1, but there were few reported cases. Over 50 cases were reported in 1994, and males outnumbered females in this year. From then on, the ratio of male to female was over 1 (Figure 5).

#### Age Distribution

**Figure 6:**
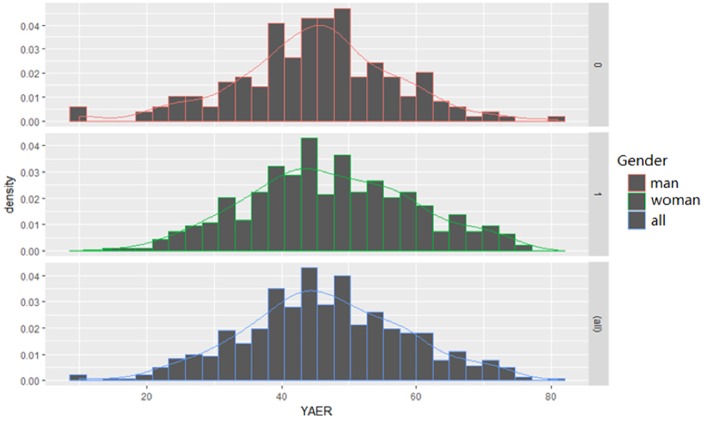
Age distribution. On the whole, the age of onset ranged from 10 to 81 years, the median age was 46 years old, the mean age was 46.39 years old (SD, 12.10 years). The age of onset in female patients ranged from 10 to 81 years, while it ranged from 15 to 75 years in male. The median age of female and male patients was same. The mean age of female and male was 44.96 years old (SD, 11.81 years) and 47.14 years old (SD, 12.20 years), respectively. Therefore, the age of onset was similar between males and females with POEMS syndrome, and the peak age for the incidence of POEMS syndrome was 46 years.

On the whole, the age of onset ranged from 10 to 81 years, the median age was 46 years old, the mean age was 46.39 years old (SD, 12.10 years). The age of onset in female patients ranged from 10 to 81 years, while it ranged from 15 to 75 years in male. The median age of female and male patients was same. The mean age of female and male was 44.96 years old (SD, 11.81 years) and 47.14 years old (SD, 12.20 years), respectively. Therefore, the age of onset was similar between males and females with POEMS syndrome, and the peak age for the incidence of POEMS syndrome was 46 years (Figure 6).

### Clinical Manifestation

#### First Symptoms

A total of 1,317 cases have reported first symptoms. The most common first symptoms were referable to peripheral neuropathy, and it was reported in 796 cases, which account for 60.44%. Followed by extravascular volume overload, and 207 (15.72%) cases reported it. The third is endocrine abnormalities which were reported in 130 (9.87%) cases. Skin changes were reported in 106 (8.05%) cases, which ranked fourth among all of the first symptoms. Organomegaly was the rarest first symptoms, just reported in 28 (2.13%) cases. The first symptoms of some patients were uncommon, such as weight loss, diarrhea, nephritis, proteinuria, and pain, which together account for 4% (Table 1).

**Table 1:** First symptoms.

**First symptoms**	**No. (1317)**	**Percentage (%)**
Peripheral neuropathy	796	60.44
Organomegaly	28	2.13
Extravascular volume overload	207	15.72
Skin changes	106	8.05
Endocrine abnormalities	130	9.87
Pain	12	0.91
Nephritis and proteinuria	8	0.61
Weight loss, diarrhea and dyspnea	30	2.28

#### Polyneuropathy

Peripheral neuropathy was reported in 1,936 patients, in which 1,049 patients had sensory-motor deficit, 318 patients had only motor deficit, 218 patients had only sensation deficit, and it was unclear in 351 patients. Among the patients with peripheral neuropathy, the initial symptom of 108 patients was motor-sensory neuron involvement, 95 patients was motor neuron involvement, 918 patients was sensory neuron involvement, and the rest of the patients were not reported in detail.

#### Organomegaly

One thousand six hundred and seventeen patients had organomegaly, in which 1,128 patients had hepatomegaly, 1,315 patients had splenomegaly, 570 patients had lymphadenopathy, and 329 patients had two or more organs enlarge among liver, spleen and lymph node. Ninety-eight patients underwent biopsy of lymph node: 54 patients had Castleman's disease. For detailed pathological types, 12 patients had plasma cell type, five patients had multicentric type, 16 patients had hyaline-vascular type, and 21 patients had unspecified type.

#### Endocrinopathy

Endocrinopathy was documented in 1,412 patients, and most involved in thyroid gland. Hypothyroidism and hyperthyroidism were reported in 770 and 32 patients, respectively. In addition, impotence and gynecomastia were common findings in men. Seven hundred and twenty-four patients had impotence, and 293 male patients had low serum testosterone level. Four hundred and seventy-six patients had gynecomastia, and 21 had nipple discharge. Moreover, dysfunction of gonadal axis and adrenocortical axis were observed in 452 and 149 patients, respectively. One hundred and sixty-nine patients had high serum prolactin levels. One hundred and fifty-one patients had irregular menses. Three hundred and ninety-six patients had diabetes mellitus or glucose intolerance. Hyperparathyroidism was not reported in this series.

#### Monoclonal Plasma Cell Dyscrasia

In present study, of the 1,077 patients who underwent serum protein electrophoresis or immunofixation electrophoresis tests at diagnosis, 685 had serum M protein.

#### Skin Changes

Skin changes were found in 1,500 patients. Hyperpigmentation was considered as the most common skin symptom in POEMS syndrome, which was reported in 1,467 patients. For other skin changes, 473 patients had hypertrichosis, 290 patients had skin thickening, 208 patients had clubbing finger, 88 patients had hyperhidrosis, 65 patients had white nail, 54 patients had skin hemangioma, 28 patients had Raynaud's phenomenon, and 18 patients had acrocyanosis.

#### Edema and Effusions

Various forms of extravascular volume overload were documented in 1,594 patients. Among them, 1,374 patients had peripheral edema, 917 patients had ascites, 677 patients had pleural effusion, 396 patients had pericardial effusion, and 91 patients had accumulation of fluid in the pelvic or perididymis.

#### Bone Lesions

A total of 846 patients had bone lesions searched. Some forms of bone lesions were documented in 372 patients, which included sclerotic bone lesions in 239 patients, lytic bone lesions in 109 patients, and mixed sclerotic and lytic components in 24 patients.

#### Others

Other clinical manifestations of POEMS syndrome were also documented in this study, which included myeloma, renal inadequacy or proteinuria, low-grade fever, pulmonary hypertension and thrombotic events in 148, 110, 102, 46, and 11 patients, respectively.

### Auxiliary Examinations

Of the 965 patients who underwent lumbar puncture, 951 had elevated cerebrospinal fluid protein level and 288 had an increase in cerebrospinal fluid pressure. Five hundred and sixty-nine of those 576 patients who had nerve conduction/electromyogram studies had peripheral neuropathy. Bone marrow aspiration was underwent in 680 patients, in which the plasma cell count of 216 patients had increased more than 2%, and 148 patients were diagnosed with myeloma. In addition, 68 patients had anemia, 51 patients had thrombocytosis, and 43 were diagnosed with polycythemia. The serum VEGF level was measured in 29 patients, in which 5 patients had obviously elevated serum VEGF level. The urine Bence-Jones protein level was measured in 601 patients, and 103 patients had Bence-Jones protein in their urine (Table 2).

**Table 2:** Auxiliary examinations.

**Auxiliary examinations**	**No. patients**	**Percentage (%)**
High CSF protein level	951	98.55
High CSF pressure level	288	29.84
Nerve conduction/electromyogram studies	569	98.78
Urine Bence-Jones protein	103	17.14
Anemia	68	10.46
Thrombocytosis	51	7.85
Elevated Bone marrow plasma cell level (more than 2%)	216	31.76
Myeloma	148	21.76
Elevated VEGF levels	5	17.24
Polycythemia	43	6.61

### Treatment, Follow-Up, and Prognosis

The data of treatment were available in 137 papers. Among those, 54 papers reported the treatment with glucocorticoid, 19 with plasma exchange, 10 with immunosuppressive agents, seven with thalidomide, 17 with bone marrow stem cell transplantation, four with Chinese traditional medicine, and 12 of 26 papers which documented the treatment with chemotherapy reported the effect of bortezomib-based chemotherapy. The data of follow-up were documented in this study. The follow-up time reported by 15 papers was over 6 months, and the longest time of follow-up was 13 years. The patients included in 20 papers were followed up until they death. Among them, the patients from five papers died of heart failure, from two papers died of pericardial effusion, from six papers died of renal failure, and from seven papers died of serious infections. The characteristics of treatment, follow-up and prognosis were not detailed in most of the studies we included. It firmly indicated that the processes of follow-up were not complete in China, and a more robust methodology will be required to assess the condition of patients of POEMS syndrome in future.

### Initially and Subsequently Acquired Features of POEMS

**Figure 7:**
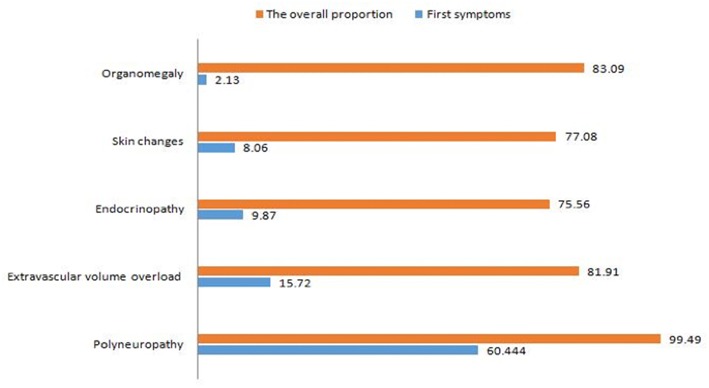
Initially and subsequently acquired features of POEMS. The prevalence of peripheral neuropathy was 99.49%, and the initial symptom of 60.44% of patients was peripheral neuropathy while 39.05% of patients had it in the development of disease. The prevalence of edema and effusions, endocrinopathy, skin changes, and organomegaly was 81.91, 75.56, 77.08, and 83.09%, respectively, and the patients with above symptoms as initial accounted for 15.72, 9.87, 8.05, and 2.13%, respectively.

In the present study, the prevalence of peripheral neuropathy was 99.49%, and the first symptom of 60.44% of patients was peripheral neuropathy while 39.05% of patients had it in the development of disease. The prevalence of edema and effusions, endocrinopathy, skin changes and organomegaly was 81.91, 75.56, 77.08, and 83.09% respectively, and the patients with above symptoms as first accounted for 15.72, 9.87, 8.05, and 2.13%, respectively (Figure 7).

## Discussions

### Epidemiological Characteristics

The ratio of male to female was 2.23, which is higher than that of 2.10 in Japan (1) or that of 1.68 in the USA (10). The median age of patients with POEMS syndrome was 46 years, which was equal to that of 46 years in Japan (1) but lower than that of 51 years in the USA (10). The age distribution of male and female was similar. In China, the number of reported cases increased in fluctuation from 1986 and peaked in 2010. The top seven provinces on the number of cases were ordinally Beijing, Shanghai, Henan, Zhejiang, Liaoning, Hebei, and Sichuan, and each province reported more than 100 cases. The possible reasons are large population and/or developed provinces. No case was included in Taiwan, Macao, Hong Kong, and Tibet. There are two studies (37, 38) in Hong Kong published in the international journals. Tibet is a developing province and difficultly recognized the disease. The top three departments of Neurology, Hematology, and Endocrinology, reported about 75% patients. In particular, department of neurology was accounted for 53.73% cases because peripheral neuropathy is the typical symptom of POEMS syndrome. In a word, the distribution of POEMS syndrome varied from area to area, and epidemiological investigations are needed for identifying it.

### Clinical Features

The prevalence of peripheral neuropathy in China (99.49%) was equal to those reported in Japan (100%) (1) and the USA (100%) (10). The most common first symptoms that brought patients to medical attention consisted of numbness and/or weakness of limbs due to peripheral neuropathy. The sensorimotor polyneuropathy was the first symptom in 60.44% of Chinese patients, which was higher than that of 51% in Japan (1). The remaining 39.5% of the Chinese patients had peripheral neuropathy in the progress of disease. These might be the causes of delayed diagnosis or even misdiagnosis. Therefore, suspected patients should be carefully observed peripheral neuropathy, no matter whether it is the first symptom or not. The neuropathy of POEMS syndrome was symmetrical and ascending. Nerve electromyogram and nerve biopsy are the useful auxiliary examinations.

The prevalence of organomegaly in China (83.09%) was higher than that of USA (50.51%) (10), whereas Japan (1) did not report the data. The most common type of organomegaly was splenomegaly in China (67.57%), hepatomegaly in Japan (78.43%) (1), and Lymphadenopathy in the USA (26.26%) (10). In addition, 55.1% of patients with Lymphadenopathy who underwent biopsy of lymph node have Castleman's disease. Patients without Castleman's disease have more serious peripheral neuropathy and induce worse prognosis than patients with Castleman's disease (39). Therefore, biopsy of lymph node is required for patients who had Lymphadenopathy.

Endocrine abnormality is a defining feature of POEMS (4). Thyroid gland was one of the most commonly involved endocrine glands in POEMS syndrome (12). The prevalence of hypothyroidism in China (39.57%) is higher than that of Japan (24%) (1) and that of the USA (14.14%) (10). Hypothyroidism may be one of the reasons leading to extravascular volume overload (40). Active and effective treatment for hypothyroidism would release those symptoms and improve overall situation of patients (41). In China, the prevalence of hyperthyroidism was 1.64% in POEMS patients, which was significantly lower than hypothyroidism. There were no reports of hyperthyroidism among POEMS patients in Nakanishi et al.'s (1) or Dispenzieri et al.'s (10, 42) study. The prevalence of glucose metabolism abnormalities in China (20.35%) was similar to that of Japan (25.49%) (1), and significantly higher than that of the USA (3.03%) (10). There were 21.8 and 46.77% patients with reduced testosterone level in present study and Dispenzieri et al.'s (10), respectively. It suggested that hypogonadism in male patients may be related to the decrease of testosterone though Japan did not report the data. Patients may conceal the symptoms, which lowers the incidence. There were 30.95% patients who had gynaecomastia or galactorrhea, and 8.68% patients who had increased serum prolactin level.

In the present study, there were 63.6% patients had a detectable monoclonal protein in their serum, which was lower than that of the USA (85%) (10) and Japan (75%) (1). Of those proteins, 42.4–59.3% were lgA type and most of time they were lambda light chains. The monoclonal protein is typically small and will be missed on serum protein electrophoresis if immunofixation is not done (43, 44). However, immunofixation electrophoresis was not commonly used in the included cases.

The prevalence of skin changes in China (77.08%) was higher than that of USA (67.68%) (10), whereas Japan (1) did not report the data. The most common abnormality was hyperpigmentation, followed by hypertrichosis. Additional common skin changes further included skin thickening, hemangiomata, and flushing.

Compared with the two previous large studies in China (12, 13), there are similar demographic characteristics including age and sex ratio, prevalence of papilledema, Castleman's disease, organomegaly, skin change, bone lesionsand, and extravascular volume overload in our study. However, as diagnosis criteria proposed by Nakanishi et al. (1) and Dispenzieri et al. (10) were used in the present study considering the data integrity, we also included patients with no report on peripheral neuropathy or detectable serum monoclonal protein in our series, which made the prevalence of peripheral neuropathy and detectable serum monoclonal protein was lower than those reported by Li et al. (12) and Wang et al. (13). In addition, the prevalence of pulmonary hypertension, thrombocytosis, polycythemia, and elevated VEGF level was lower than those reported by Li et al. (12) and Wang et al. (13). The subjects included in their studies were the patients who was admitted to the same hospital in China. Those patients received all kinds of auxiliary examinations and provided integrated data. The data of auxiliary examinations in some of our included studies was not with great concern and not be reported integrally.

### Diagnosis

The diagnosis is often delayed when clinicians fail to recognize the diagnostic features outside the immediate sphere of knowledge of their specialty, as each major feature of POEMS is relevant to disparate medical specialties (45). In recent years, revised diagnostic criteria have been published by Dispenzieri et al. (5, 10). By definition, the diagnosis of POEMS syndrome is confirmed when both the polyneuropathy and monoclonal plasma cell disorder are present in association with one of the other three major criteria: Castleman disease, sclerotic bone lesions, and vascular endothelial growth factor elevation, and one of the six minor criteria: organomegaly, extravascular volume overload, endocrinopathy, skin changes, papilledema, thrombocytosis, or, polycythemia. In the present study, all the included patients met the diagnostic criteria proposed by Nakanishi et al. (1) or Dispenzieri et al. (10).

Making a diagnosis of POEMS is a challenge. However, a complete medical history and physical examination followed by appropriate testing, may be required to distinguish POEMS syndrome from other conditions such as chronic inflammatory demyelinating polyneuropathy (CIDP), monoclonal gammopathy of undetermined significance (MGUS) neuropathy and immunoglobulin light-chain amyloid neuropathy (5). These tests include serum and urine protein electrophoresis and immunofixation, CT scans, radiographic assessment of bones, careful analysis of a bone marrow biopsy, and measurement of serum and/or plasma VEGF (25, 46–49). Testing of plasma or serum VEGF levels is useful both for diagnosing and monitoring the disease (16, 18, 20, 48, 50). The sensitivity of elevated serum VEGF for the diagnosis of POEMS was 100% as well as its specificity was above 90% (51). Any patient considered as suffer CIDP should be considered as a possible POEMS syndrome patient if they are not responding to standard CIDP therapy, and additional testing should be done to rule in or rule out the diagnosis of POEMS (5). POEMS syndrome should be taken into consideration when patients had these clinical presentations of unknown cause including peripheral neuropathy, monoclonal protein (especially lambda light chain), thrombocytosis, anasarca, papilledemarefractory ascites, peripheral edema, gynecomastia, skin changes, organomegaly, and cerebrovascular disease (52–54).

### Treatment

Treatment algorithm of POEMS syndrome is directed at the underlying monoclonal plasma cell disorder and depends on the extent of the skeletal lesions and bone marrow involvement. In the case of patients with two or fewer bone lesion without clonal plasma cells found on iliac crest biopsy, radiation is the first-line therapy. Systemic therapy should be applied on patients with generalized disease, characterized by either bone diffuse marrow involvement and/or more than three skeletal lesions (5, 26). High-dose chemotherapy with peripheral blood stem cell transplant is the current gold standard treatment for POEMS. This treatment strategy shows good hematological control, neurological response, along with the improvement of the neuropathy (55, 56). Treatment-related morbidity and mortality can be minimized with engraftment-type syndrome recognized and treated, leading to good survival (57). Alkylator-based therapy was also a good choice for POEMS. Melphalan in combination with dexamethasone resulted in high response in patients with POEMS syndrome (12). Immunomodulatory agents such as thalidomide and lenalidomide are candidates to be treatment options for POEMS syndrome because they excellently suppressed the proliferation of monoclonal plasma cell as well as anti-VEGF and anti-cytokines (21, 58–60). Notably, lenalidomide is better than thalidomide considering the risk of a peripheral neuropathy (61, 62). Similar to lenalidomide and thalidomide, bortezomib is also effective in POEMS syndrome (63, 64).

The treatments of POEMS syndrome were comprehensive in our included studies. Glucocorticoid and chemotherapy were the mostly common therapies adopted in included patients while no patients received radiation therapy. It may relate to the low incidence of local bone lesion along with low response rate and high cost of radiation therapy in China (12). Development of novel interventions is needed because of a lack of standard therapeutics for the disease.

### Survival

The median survival time was only 5–7 years in China if without successful treatment (12), though it was demonstrated that 10 years survival rate of this disease was 62% based on the Mayo Clinic experience (3). The most common causes of death are cardiorespiratory failure, progressive inanition, infection, capillary leak-like syndrome, and renal failure. But the exact relationship between above symptoms and prognosis of the disease is still not fully clear. Kourelis et al. (3) demonstrated that the three factors associated with superior overall survival were younger age, albumin >3.2 g/dL and attainment of complete hematologic response. Wang et al. (13) have developed and validated a prognostic nomogram containing four baseline clinical variables that could evaluate individualized prognosis in patients with newly diagnosed POEMS syndrome. This prognostic system for overall survival has been validated by a mayo clinic cohort and was proposed to be applied to a broader patient population (65). In the present study, seldom case reported the survival, and thus the epidemiology of survival rate of this disease is needed in the future.

## Limitations

The present study has some limitations. The quality of retrospective study was depended on the included published literature. Although we systematically searched the Chinese databases, we may have missed qualified literature that published in other language journals. Many POEMS syndrome patients may be diagnosed but they were not reported, which could lower the incidence of the disease. In addition, the diagnosis criteria proposed by (1) and (10) were used in the present study. As the diagnosis criteria of POEMS syndrome is developing along with the changes of time, there was a leading diagnosis criterion in every stage of medical development. Physicians always make diagnosis according to the leading diagnosis criteria in their era. It will be more acceptable to use the diagnosis criteria published by A Dispenzieri. However, the eligible patients diagnosed based on the diagnosis criteria published by Nankanish would missed, which was inconsistent with our purpose to include extensive and comprehensive data. Using the eligibility from the Nakanishi paper from 1984 is a deliberate decision after weighing the advantages and disadvantages. In China, the quality of medical service that patients received in different economic level region is different, and the understanding of POEMS syndrome improved along with time. This makes the comprehensive data more acceptable for reflecting the real situation of this disease in China than the selected data. It is also difficult to re-diagnosed the patients met the Nakanishi's diagnosis criteria with diagnosis criteria proposed by A Dispenzieri because there is no enough information in many primary studies. Furthermore, as the data of auxiliary examinations was not with great concern and not be reported integrally in many included case reports. The great efforts we made to collect the relevant data cannot cover the limitation of the primary studies. Because of this limitation, the prevalence of monoclonal protein, pulmonary hypertension, thrombocytosis, polycythemia, and elevated VEGF level was lower than those reported in other Chinese series. For example, as the immunofixation electrophoresis was not commonly available in the past in China, only about half of the patients in this series were screened for monoclonal protein.

## Conclusion

In China, males with POEMS syndrome were 2.23 times as females patients. The age of onset was similar between males and females, and the peak age for the incidence of POEMS syndrome was 46 years. Most of the patients were reported by the department of neurology, hematology, and endocrinology. The most common initial symptoms were referable to peripheral neuropathy, followed by extravascular volume overload, endocrine abnormalities, skin changes, and organomegaly. Peripheral neuropathy was present in almost all of patients while extravascular volume overload, endocrine abnormalities, skin changes, and organomegaly were present in the majority of patients in the development of disease processes. Given the rarity of POEMS syndrome and difficulty of clinical diagnosis, a comprehensive database of POEMS patients is required in the future. In order to assess efficacy of therapies and recommend most effective treatment strategy by meta-analysis, standardized outcome measures that accurately reflect disease severity and change are urgently needed (66).

## Author Contributions

YoW, L-BH, G-QZ, and YaW designed the study. L-BH, Y-HS, HF, and ZX participated in the collection and analysis the data. YoW wrote the manuscript. YaW and G-QZ revised the study. YoW and L-BH generated the figures. All authors provided the approval of the final manuscript for submission.

### Conflict of Interest Statement

The authors declare that the research was conducted in the absence of any commercial or financial relationships that could be construed as a potential conflict of interest.
